# (2-Chloro­benzo[*h*]quinolin-3-yl)methanol

**DOI:** 10.1107/S1600536810010767

**Published:** 2010-03-27

**Authors:** F. Nawaz Khan, S. Mohana Roopan, Venkatesha R. Hathwar, R. Rajesh, M. Khawar Rauf

**Affiliations:** a Chemistry Division, School of Advanced Sciences, VIT University, Vellore 632 014, Tamil Nadu, India; b Solid State and Structural Chemistry Unit, Indian Institute of Science, Bangalore 560 012, Karnataka, India; c Department of Chemistry, Bharathiar University, Coimbatore, Tamil Nadu, India; d Department of Chemistry, Quaid-i-Azam University Islamabad, 45320 Pakistan

## Abstract

In the title mol­ecule, C_14_H_10_ClNO, all non-H atoms are coplanar (r.m.s deviation = 0.0266 Å). In the crystal, symmetry-related mol­ecules are hydrogen bonded *via* inter­molecular O—H⋯O inter­actions, forming chains along the *b* axis.

## Related literature

The title compound was obtained by the reduction of an aldehyde using Montmorillonite K-10 as catalyst. For background to the use of Montmorillonite clays as catalysts, see: Roopan *et al.* (2009*b* ▶). For related structures, see: Khan *et al.* (2010*a* ▶,*b* ▶); Roopan *et al.* (2009*a* ▶).
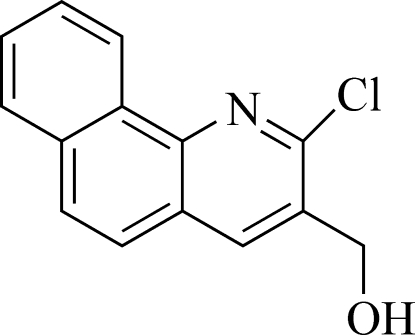


## Experimental

### 

#### Crystal data

C_14_H_10_ClNO
*M* *_r_* = 243.68
Monoclinic, 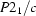

*a* = 16.6953 (4) Å
*b* = 4.61459 (11) Å
*c* = 14.5588 (3) Å
β = 95.123 (2)°
*V* = 1117.16 (5) Å^3^
*Z* = 4
Mo *K*α radiation
μ = 0.32 mm^−1^
*T* = 295 K
0.35 × 0.30 × 0.28 mm

#### Data collection

Oxford Diffraction Xcalibur diffractometer
Absorption correction: multi-scan (*CrysAlis PRO*; Oxford Diffraction, 2009 ▶) *T*_min_ = 0.896, *T*_max_ = 0.915
11643 measured reflections
2200 independent reflections
1717 reflections with *I* > 2σ(*I*)
*R*_int_ = 0.028

#### Refinement

*R*[*F* ^2^ > 2σ(*F* ^2^)] = 0.034
*wR*(*F* ^2^) = 0.093
*S* = 1.08
2200 reflections
155 parameters
H-atom parameters constrained
Δρ_max_ = 0.19 e Å^−3^
Δρ_min_ = −0.22 e Å^−3^

### 

Data collection: *CrysAlis PRO* (Oxford Diffraction, 2009 ▶); cell refinement: *CrysAlis PRO*; data reduction: *CrysAlis PRO*; program(s) used to solve structure: *SHELXS97* (Sheldrick, 2008 ▶); program(s) used to refine structure: *SHELXL97* (Sheldrick, 2008 ▶); molecular graphics: *ORTEP-3 for Windows* (Farrugia, 1997 ▶); software used to prepare material for publication: *WinGX* (Farrugia, 1999 ▶).

## Supplementary Material

Crystal structure: contains datablocks I, global. DOI: 10.1107/S1600536810010767/pv2269sup1.cif

Structure factors: contains datablocks I. DOI: 10.1107/S1600536810010767/pv2269Isup2.hkl

Additional supplementary materials:  crystallographic information; 3D view; checkCIF report

## Figures and Tables

**Table 1:** Hydrogen-bond geometry (Å, °)

*D*—H⋯*A*	*D*—H	H⋯*A*	*D*⋯*A*	*D*—H⋯*A*
O1—H1⋯O1^i^	0.82	1.90	2.7154 (12)	175

Symmetry code: (i) 
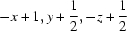
.

**Fig. 1.**
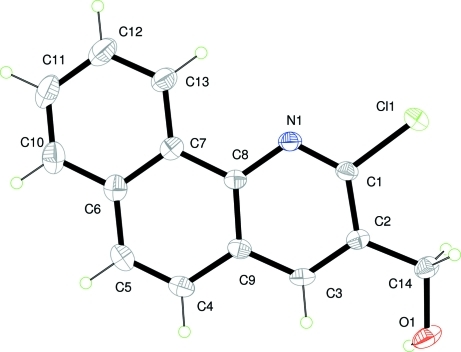
Molecular structure of (I) showing atom numbering scheme. Displacement ellipsoids are drawn at the 30% probability level.

## supplementary crystallographic information

### Comment

Montmorillonite clays have been found to effectively catalyze a broad range of chemical reactions (Roopan *et al.*, 2009*b*). In continuation of our green chemical approach on the structural chemistry of disubstituted quinolines (Khan *et al.*, 2010*a*,*b*; Roopan *et al.*, 2009*a*), we have demonstarted the reduction of an aldehyde using Montmorillonite K-10 as a catalyst, to obtain the title alcohol. In this article, the crystal structure of the title molecule is presented.

In the title molecule (Fig. 1) all non-hydrogen atoms are coplanar (r.m.s deviation = 0.0266 Å); the C—C—C—O torsion angles are -0.9 (2) and -179.73 (13)°. The crystal structure is composed of discrete molecules with bond lengths and angles quite typical for compounds of this class and agree well with the corresponding bond lengths and angles reported for some related compounds (Khan *et al.*, 2010*a* & 2010*b*; Roopan *et al.*, 2009). In the crystal, symmetry related molecules are hydrogen bonded *via* intermolecular O—H···O type interactions forming one dimensional chains along the *b*-axis. In addition, an intramolecular interaction, C3—H3···O1 further consolidated the crystal structure.

### Experimental

2-Chlorbenzo[h]quinoline-3-carbaldehyde (241 mg, 1 mmol), sodium borohydride (38 mg, 1 mmol) and a catalytic amount of montmorillonite K-10 (100 mg) were placed in a beaker. The contents were irradiated at 500 W for 5 min. The product was dissolved in ethyl acetate and the residue removed by filtration. The filtrate was subjected to column chromatography on silica, and ethyl acetate/petroleum ether was used as the eluant. The solvent was evaporated and the residue recrystallized from chloroform to give colorless crystals.

### Refinement

Hydrogen atoms were placed in calculated positions (C—H 0.93–0.97 Å, O—H 0.82 Å)and were included in the refinement in the riding model approximation, with U_iso_(H) set to 1.2–1.5U_eq_(C,*O*).

### Crystal data

**Table:**

C_14_H_10_ClNO	*F*(000) = 504
*M**_r_* = 243.68	*D*_x_ = 1.449 Mg m^−^^3^
Monoclinic, *P*2_1_/*c*	Mo *K*α radiation, λ = 0.71073 Å
Hall symbol: -P 2ybc	Cell parameters from 11643 reflections
*a* = 16.6953 (4) Å	θ = 2.5–26.0°
*b* = 4.61459 (11) Å	µ = 0.32 mm^−^^1^
*c* = 14.5588 (3) Å	*T* = 295 K
β = 95.123 (2)°	Block, colourless
*V* = 1117.16 (5) Å^3^	0.35 × 0.30 × 0.28 mm
*Z* = 4	

### Data collection

**Table:**

Oxford Diffraction Xcalibur diffractometer	2200 independent reflections
Radiation source: fine-focus sealed tube	1717 reflections with *I* > 2σ(*I*)
graphite	*R*_int_ = 0.028
ω scans	θ_max_ = 26.0°, θ_min_ = 2.5°
Absorption correction: multi-scan (*CrysAlis PRO*; Oxford Diffraction, 2009)	*h* = −20→20
*T*_min_ = 0.896, *T*_max_ = 0.915	*k* = −5→5
11643 measured reflections	*l* = −17→17

### Refinement

**Table:**

Refinement on *F*^2^	Primary atom site location: structure-invariant direct methods
Least-squares matrix: full	Secondary atom site location: difference Fourier map
*R*[*F*^2^ > 2σ(*F*^2^)] = 0.034	Hydrogen site location: inferred from neighbouring sites
*wR*(*F*^2^) = 0.093	H-atom parameters constrained
*S* = 1.08	*w* = 1/[σ^2^(*F*_o_^2^) + (0.0447*P*)^2^ + 0.1644*P*] where *P* = (*F*_o_^2^ + 2*F*_c_^2^)/3
2200 reflections	(Δ/σ)_max_ = 0.001
155 parameters	Δρ_max_ = 0.19 e Å^−^^3^
0 restraints	Δρ_min_ = −0.22 e Å^−^^3^

### Special details

**Table:**

Geometry. All esds (except the esd in the dihedral angle between two l.s. planes) are estimated using the full covariance matrix. The cell esds are taken into account individually in the estimation of esds in distances, angles and torsion angles; correlations between esds in cell parameters are only used when they are defined by crystal symmetry. An approximate (isotropic) treatment of cell esds is used for estimating esds involving l.s. planes.
Refinement. Refinement of *F*^2^ against ALL reflections. The weighted *R*-factor wR and goodness of fit *S* are based on *F*^2^, conventional *R*-factors *R* are based on *F*, with *F* set to zero for negative *F*^2^. The threshold expression of *F*^2^ > σ(*F*^2^) is used only for calculating *R*-factors(gt) etc. and is not relevant to the choice of reflections for refinement. *R*-factors based on *F*^2^ are statistically about twice as large as those based on *F*, and *R*- factors based on ALL data will be even larger.

### Fractional atomic coordinates and isotropic or equivalent isotropic displacement parameters (Å^2^)

**Table:**

	*x*	*y*	*z*	*U*_iso_*/*U*_eq_	
Cl1	0.38036 (3)	0.30225 (12)	0.55246 (3)	0.05783 (19)	
N1	0.28031 (8)	0.6236 (3)	0.45110 (8)	0.0350 (3)	
O1	0.47139 (8)	0.0886 (3)	0.28368 (8)	0.0497 (3)	
H1	0.4911	0.2355	0.2633	0.074*	
C2	0.37487 (9)	0.3556 (3)	0.36689 (10)	0.0315 (3)	
C1	0.33981 (9)	0.4434 (3)	0.44662 (10)	0.0331 (4)	
C7	0.18191 (9)	0.9418 (4)	0.37325 (11)	0.0366 (4)	
C9	0.27796 (9)	0.6741 (3)	0.28570 (10)	0.0333 (4)	
C3	0.34176 (9)	0.4759 (3)	0.28642 (10)	0.0339 (4)	
H3	0.3619	0.4254	0.2311	0.041*	
C4	0.24384 (10)	0.8084 (4)	0.20323 (11)	0.0426 (4)	
H4	0.2637	0.7635	0.1472	0.051*	
C8	0.24801 (9)	0.7420 (3)	0.37074 (10)	0.0309 (3)	
C6	0.14987 (10)	1.0709 (4)	0.28978 (12)	0.0417 (4)	
C13	0.14771 (10)	1.0095 (4)	0.45516 (12)	0.0478 (4)	
H13	0.1685	0.9266	0.5105	0.057*	
C5	0.18321 (11)	0.9994 (4)	0.20550 (12)	0.0477 (5)	
H5	0.1625	1.0872	0.1510	0.057*	
C14	0.44481 (9)	0.1482 (4)	0.37137 (11)	0.0395 (4)	
H14A	0.4891	0.2296	0.4108	0.047*	
H14B	0.4291	−0.0321	0.3990	0.047*	
C10	0.08499 (11)	1.2661 (4)	0.29225 (15)	0.0551 (5)	
H10	0.0639	1.3552	0.2381	0.066*	
C11	0.05304 (11)	1.3253 (5)	0.37239 (16)	0.0638 (6)	
H11	0.0100	1.4531	0.3726	0.077*	
C12	0.08390 (12)	1.1971 (5)	0.45434 (15)	0.0609 (6)	
H12	0.0612	1.2386	0.5089	0.073*	

### Atomic displacement parameters (Å^2^)

**Table:**

	*U*^11^	*U*^22^	*U*^33^	*U*^12^	*U*^13^	*U*^23^
Cl1	0.0679 (3)	0.0734 (4)	0.0331 (2)	0.0227 (3)	0.0093 (2)	0.0130 (2)
N1	0.0384 (7)	0.0381 (8)	0.0294 (7)	0.0009 (6)	0.0085 (5)	−0.0015 (6)
O1	0.0613 (8)	0.0343 (7)	0.0590 (8)	0.0033 (6)	0.0365 (6)	−0.0016 (6)
C2	0.0339 (8)	0.0279 (8)	0.0340 (8)	−0.0049 (7)	0.0095 (6)	−0.0023 (6)
C1	0.0390 (8)	0.0338 (9)	0.0274 (8)	−0.0005 (7)	0.0076 (6)	0.0018 (7)
C7	0.0335 (8)	0.0339 (9)	0.0424 (9)	−0.0040 (7)	0.0040 (7)	−0.0054 (7)
C9	0.0355 (8)	0.0351 (9)	0.0297 (8)	−0.0068 (7)	0.0049 (6)	−0.0011 (7)
C3	0.0388 (8)	0.0368 (9)	0.0277 (8)	−0.0058 (7)	0.0122 (6)	−0.0062 (7)
C4	0.0469 (10)	0.0511 (11)	0.0299 (8)	−0.0052 (9)	0.0047 (7)	−0.0014 (8)
C8	0.0316 (8)	0.0321 (8)	0.0293 (7)	−0.0040 (6)	0.0051 (6)	−0.0030 (6)
C6	0.0380 (9)	0.0358 (9)	0.0500 (10)	−0.0046 (7)	−0.0034 (7)	−0.0026 (8)
C13	0.0440 (10)	0.0516 (11)	0.0484 (10)	0.0057 (9)	0.0075 (8)	−0.0106 (8)
C5	0.0511 (10)	0.0491 (11)	0.0410 (9)	−0.0024 (9)	−0.0062 (8)	0.0071 (8)
C14	0.0422 (9)	0.0354 (10)	0.0429 (9)	0.0008 (7)	0.0145 (7)	0.0001 (7)
C10	0.0465 (11)	0.0458 (11)	0.0697 (13)	0.0043 (9)	−0.0130 (9)	−0.0032 (10)
C11	0.0427 (11)	0.0594 (13)	0.0874 (16)	0.0163 (10)	−0.0053 (10)	−0.0185 (12)
C12	0.0467 (11)	0.0668 (14)	0.0698 (13)	0.0104 (10)	0.0094 (9)	−0.0210 (11)

### Geometric parameters (Å, °)

**Table:**

Cl1—C1	1.7525 (15)	C4—C5	1.345 (2)
N1—C1	1.3014 (19)	C4—H4	0.9300
N1—C8	1.3585 (19)	C6—C10	1.412 (2)
O1—C14	1.4155 (19)	C6—C5	1.430 (2)
O1—H1	0.8200	C13—C12	1.372 (2)
C2—C3	1.368 (2)	C13—H13	0.9300
C2—C1	1.405 (2)	C5—H5	0.9300
C2—C14	1.507 (2)	C14—H14A	0.9700
C7—C13	1.402 (2)	C14—H14B	0.9700
C7—C6	1.415 (2)	C10—C11	1.353 (3)
C7—C8	1.441 (2)	C10—H10	0.9300
C9—C3	1.403 (2)	C11—C12	1.389 (3)
C9—C8	1.411 (2)	C11—H11	0.9300
C9—C4	1.424 (2)	C12—H12	0.9300
C3—H3	0.9300		
			
C1—N1—C8	117.39 (13)	C10—C6—C5	121.83 (17)
C14—O1—H1	109.5	C7—C6—C5	119.57 (16)
C3—C2—C1	115.09 (14)	C12—C13—C7	120.52 (18)
C3—C2—C14	123.18 (14)	C12—C13—H13	119.7
C1—C2—C14	121.71 (14)	C7—C13—H13	119.7
N1—C1—C2	126.98 (14)	C4—C5—C6	121.58 (16)
N1—C1—Cl1	115.47 (11)	C4—C5—H5	119.2
C2—C1—Cl1	117.54 (12)	C6—C5—H5	119.2
C13—C7—C6	119.02 (16)	O1—C14—C2	112.85 (13)
C13—C7—C8	122.33 (15)	O1—C14—H14A	109.0
C6—C7—C8	118.64 (15)	C2—C14—H14A	109.0
C3—C9—C8	117.80 (13)	O1—C14—H14B	109.0
C3—C9—C4	122.41 (14)	C2—C14—H14B	109.0
C8—C9—C4	119.78 (15)	H14A—C14—H14B	107.8
C2—C3—C9	121.29 (14)	C11—C10—C6	120.88 (18)
C2—C3—H3	119.4	C11—C10—H10	119.6
C9—C3—H3	119.4	C6—C10—H10	119.6
C5—C4—C9	120.65 (16)	C10—C11—C12	120.66 (18)
C5—C4—H4	119.7	C10—C11—H11	119.7
C9—C4—H4	119.7	C12—C11—H11	119.7
N1—C8—C9	121.44 (14)	C13—C12—C11	120.30 (19)
N1—C8—C7	118.79 (13)	C13—C12—H12	119.8
C9—C8—C7	119.77 (13)	C11—C12—H12	119.8
C10—C6—C7	118.61 (17)		
			
C8—N1—C1—C2	−0.2 (2)	C6—C7—C8—N1	178.27 (14)
C8—N1—C1—Cl1	178.89 (11)	C13—C7—C8—C9	177.63 (15)
C3—C2—C1—N1	0.1 (2)	C6—C7—C8—C9	−1.5 (2)
C14—C2—C1—N1	178.96 (15)	C13—C7—C6—C10	0.7 (2)
C3—C2—C1—Cl1	−178.97 (11)	C8—C7—C6—C10	179.84 (15)
C14—C2—C1—Cl1	−0.1 (2)	C13—C7—C6—C5	−178.81 (16)
C1—C2—C3—C9	0.6 (2)	C8—C7—C6—C5	0.4 (2)
C14—C2—C3—C9	−178.32 (14)	C6—C7—C13—C12	0.3 (3)
C8—C9—C3—C2	−1.0 (2)	C8—C7—C13—C12	−178.85 (16)
C4—C9—C3—C2	178.28 (15)	C9—C4—C5—C6	−1.2 (3)
C3—C9—C4—C5	−179.35 (15)	C10—C6—C5—C4	−178.47 (16)
C8—C9—C4—C5	0.0 (2)	C7—C6—C5—C4	1.0 (3)
C1—N1—C8—C9	−0.4 (2)	C3—C2—C14—O1	−0.9 (2)
C1—N1—C8—C7	179.86 (14)	C1—C2—C14—O1	−179.73 (13)
C3—C9—C8—N1	0.9 (2)	C7—C6—C10—C11	−1.1 (3)
C4—C9—C8—N1	−178.40 (14)	C5—C6—C10—C11	178.35 (18)
C3—C9—C8—C7	−179.28 (13)	C6—C10—C11—C12	0.6 (3)
C4—C9—C8—C7	1.4 (2)	C7—C13—C12—C11	−0.8 (3)
C13—C7—C8—N1	−2.6 (2)	C10—C11—C12—C13	0.4 (3)

### Hydrogen-bond geometry (Å, °)

**Table:**

*D*—H···*A*	*D*—H	H···*A*	*D*···*A*	*D*—H···*A*
O1—H1···O1^i^	0.82	1.90	2.7154 (12)	175
C3—H3···O1	0.93	2.47	2.809 (2)	102

Symmetry codes: (i) −*x*+1, *y*+1/2, −*z*+1/2.
